# Analysis of the Involvement of NMDA Receptors in Analgesia and Hypothermia Induced by the Activation of TRPV1 Ion Channels

**DOI:** 10.32607/actanaturae.11829

**Published:** 2023

**Authors:** E. A. Ivanova, A. I. Matyushkin, T. A. Voronina

**Affiliations:** V.V. Zakusov Research Institute of Pharmacology, Moscow, 125315 Russian Federation

**Keywords:** NMDA receptors, TRPV1 ion channels, capsaicin, mice, nociception, thermoregulation

## Abstract

NMDA glutamate receptors play an important role in normal and pathophysiological nociception. At the periphery, they can interact with TRPV1 ion channels. The blockade of TRPV1 ion channels decreases NMDA-induced hyperalgesia, and NMDA receptor antagonists suppress the pain response to the TRPV1 agonist capsaicin. Since TRPV1 ion channels and NMDA receptors can functionally interact at the periphery, it would be interesting to investigate the possibility that they interact in the CNS. A single subcutaneous injection of 1 mg/kg of capsaicin was found to raise the thermal pain threshold in the tail flick test in mice, which reproduces the spinal flexion reflex, owing to the ability of capsaicin to cause long-term desensitization of nociceptors. Preventive administration of either noncompetitive NMDA receptor antagonists (high-affinity MK-801 20 μg/kg and 0.5 mg/kg subcutaneously; low-affinity hemantane 40 mg/kg intraperitoneally) or the selective TRPV1 antagonist BCTC (20 mg/kg intraperitoneally) inhibit the capsaicin-induced increase in the pain threshold. Capsaicin (1 mg/kg, subcutaneous injection) induces transient hypothermia in mice, which is brought about by hypothalamus-triggered vegetative reactions. This effect is prevented by BCTC but not by the noncompetitive NMDA receptor antagonists.

## INTRODUCTION

The interaction between glutamate and glutamate receptors is central for excitatory transmission in the central nervous system (CNS) and plays a crucial role in normal and pathophysiological nociception. In particular, the long-term activation of nociceptors, induced by either damage to tissues and nerves or inflammation, leads to a continuous release of glutamate; together with released neuropeptides, this may cause prolonged membrane depolarization, might eliminate the voltage-gated block of the ion channel of NMDA glutamate receptors by magnesium, and ensure their activation [1]. NMDA glutamate receptors are located on primary afferents [2, 3, 4, 5], and their stimulation leads to nociceptor activation or sensitization [2, 6, 7, 8, 9]. At the periphery, NMDA glutamate receptors can interact with TRPV1 ion channels in the calcium/calmodulin-dependent protein kinase type II (CaMKII) and protein kinase C (PKC) pathways; administration of AMG9810, an antagonist of TRPV1 ion channels, suppresses NMDA (N-methyl-D-aspartic acid)-induced mechanical hyperalgesia in rats [10]. Injection of antagonists of ionotropic NMDA and AMPA or metabotropic mGluR1 glutamate receptors into the plantar surface in rats decreases thermal hyperalgesia induced by capsaicin, a TRPV1 ion channel agonist, and inhibits the elevation of the glutamate level in subcutaneous perfusate observed after the injection of capsaicin into the animals’ metatarsal region [11]. Noncompetitive antagonists of NMDA receptors (high-affinity (5S,10R)-(+)-5-methyl-10,11-dihydro- 5H-dibenzo[a,d]cyclohepten-5,10-imine hydrogen maleate (MK-801) and low-affinity N-(2-adamantyl)- hexamethyleneimine hydrochloride (hemantane)) reduce the duration of the pain response to the sub cutaneous injection of a capsaicin solution into the metatarsal region of mice when applied dermally, systemically (intraperitoneally for hemantane and subcutaneously for MK-801), and via the subcutaneous intraplantar route [12].

TRPV1 ion channels are voltage-gated non-selective cation channels that are expressed by primary afferent neurons, activated by vanilloids, low pH values (pH < 6.5), changes in osmolarity, arachidonic acid metabolites, endocannabinoids, as well as temperatures above 42°C [13, 14, 15, 16, 17], and are regarded as a potential signal integrator under pathological conditions (in particular, that is indicated by the possibility of their functional interaction with NMDA glutamate receptors in trigeminal afferent neurons in mechanical hyperalgesia [10]). Like NMDA glutamate receptors, TRPV1 ion channels are abundant in the CNS [17]. Taking into account the ability of TRPV1 ion channels and NMDA glutamate receptors to functionally interact at the periphery, it is rather interesting to study their interplay in the CNS.

The aim of this work was to evaluate the ability of noncompetitive antagonists of NMDA receptors (the high-affinity MK-801 and low-affinity hemantane) to influence the effects of capsaicin, an agonist of TRPV1 ion channels, at the CNS level: alter the pain response threshold in the tail flick test and rectal temperature in mice. The effect of the antagonists of NMDA receptors was compared to the selective antagonist of TRPV1 ion channels, 4-(3-chloro-2-pyridinyl)-N-[4- (1,1-dimethylethyl)phenyl]-1-piperazinecarboxamide (BCTC), which is capable of penetrating the blood– brain barrier (BBB) [18].

## MATERIALS AND METHODS

**Animals**

Mature male ICR mice (weight, 23–26 g) procured from the Stolbovaya husbandry of laboratory animals, Research Center of Biomedical Technologies, Federal Medical and Biological Agency (Moscow Region, Russia), were used in this study. The animals were handled in compliance with the State Standard GOST 33216-2014 "Guidelines for Accommodation and Care of Laboratory Animals. Species-Specific Provisions for Laboratory Rodents and Rabbits," State Standard GOST 33215-2014 "Guidelines for Accommodation and Care of Animals. Environment, Housing and Management," and Directive 2010/63/EU of the European Parliament and the Council of the European Union, dated September 9, 2010, on the protection of animals used for scientific purposes. Experiment conduct was approved by the Biomedical Ethics Commission of the Zakusov Research Institute of Pharmacology (Protocol No. 01 dated January 28, 2022).

**Study objects, doses, and administration routes**

NMDA receptor antagonists were the noncompetitive high-affinity antagonist (5S,10R)-(+)-5-methyl- 10,11-dihydro-5H-dibenzo[a,d]cyclohepten-5,10-imine hydrogen maleate (MK-801; Sigma Aldrich, USA) and noncompetitive low-affinity antagonist N-(2-adamantyl)-hexamethyleneimine hydrochloride (hemantane; synthesized and provided by the Chemical–Technological Laboratory of the Zakusov Research Institute of Pharmacology). The antagonist of TRPV1 ion channels was 4-(3-chloro-2-pyridinyl)- N-[4-(1,1-dimethylethyl)phenyl]-1-piperazinecarboxamide (BCTC; Sigma Aldrich, USA). The agents were administered 30 min prior to injecting the capsaicin solution: MK-801 was injected subcutaneously at doses of 20 μg/kg and 0.5 mg/kg; hemantane was injected intraperitoneally at a dose of 40 mg/kg; and BCTC was injected intraperitoneally at a dose of 20 mg/kg.

The TRPV1 ion channel agonist capsaicin (Sigma Aldrich, USA), diluted in a saline–ethanol mixture (9:1, v/v), was injected subcutaneously at a dose causing transient hypothermia in mice (1 mg/kg) [14].

**Tail flick test**

The tail flick test is based on the spinal flexion reflex in response to a progressively increasing thermal radiation stimulation of skin and is widely used for assessing the analgesic effect of various agents [19, 20]. Thermoreceptors, C- and Ad-fibers of polymodal nociceptors, and high-threshold mechanoreceptors are sequentially activated in this test. Local pain stimulation of the tail was induced by thermal radiation using a TSE-system analgesiometer (Germany). Stimulation intensity was 27%, which corresponded to a gradual temperature increase ranging from 51 to 61°C during 15 s. The latent period (LP) until tail withdrawal (15 s) was considered the maximum possible time of stimulation. The maximum possible effect (MPE) was calculated using the formula:

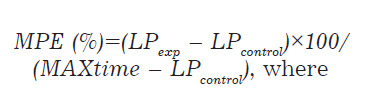



L_Pexp_ was the latent period of response in mice 30 min after administering the capsaicin solution or NMDA receptor and TRPV1 ion channel antagonists;

L_Pcontrol_ was the latent period for mice in the control group that received the solvent; and

MAXtime was the maximum possible time of stimulation (15 s).

The experimental investigation of the effect of noncompetitive NMDA receptor antagonists on changes in the pain response threshold in the tail flick test induced by capsaicin (a TRPV1 ion channel agonist) involved two stages. The influence of BCTC, a TRPV1 ion channel antagonist, and NMDA receptor antagonists on the sensitivity to thermal stimulation of the mouse tail was assessed 30 min after their administration at the first stage. At the second stage, their effect on the pain response threshold in the animals, increased by capsaicin administration, was evaluated 30 min after injecting the TRPV1 ion channel agonist. Mice that had subcutaneously received an equivalent volume (10 mL/kg) of solvents were used as control groups. Saline was used as a solvent in the first experiment. In the second experiment, saline was employed as a solvent for BCTC, hemantane, and MK-801, and a saline–ethanol mixture (9:1, v/v) was used for capsaicin; in other words, the animals received saline instead of BCTC, hemantane, or MK-801, and a saline–ethanol mixture (9:1, v/v) instead of capsaicin.

**Rectal temperature **in mice was measured using a digital rectal thermometer (Kent Scientific Corp., USA). Groups of animals that had received solvents (saline and saline–ethanol mixture (9:1, v/v)), saline and capsaicin, and groups of animals that received capsaicin 30 min after administration of the tested antagonists of NMDA receptors and TRPV1 ion channels were included in the experiment. The effects of antagonists of NMDA receptors and TRPV1 ion channels on the rectal temperature in mice that had received saline only were also compared. The rectal temperature was measured prior to injecting the saline, capsaicin, NMDA receptor antagonists, and BCTC, and every 30 min after administration of the solvent, NMDA antagonists, BCTC, and capsaicin (2 h), or every 30 min after administration of capsaicin (when injected together with NMDA receptor antagonists and BCTC (2 h)).

**Statistical analysis **of the experimental data was carried out using the Statistica 10.0 software. Data were checked for normal distribution using the Shapiro–Wilk test, followed by an evaluation of intergroup equality using the Levene’s test. For normal distribution in the groups and the homogeneity of intergroup variance, further statistical analysis was performed using a one-way analysis of variance (ANOVA), followed by group comparison using the Newman–Keuls test. The Kruskal–Wallis test, a nonparameteric alternative to one-way ANOVA, was used in the case of non-normal distribution. If statistically significant intergroup differences were detected using the Kruskal–Wallis test, we carried out pairwise comparison of samples using the Mann– Whitney U test. Intergroup differences were considered statistically significant at p < 0.05. The figures were created using the GraphPad Prism V. 8.4.3 software.

## RESULTS AND DISCUSSION

Expression of TRPV1 ion channels is maximal in the dorsal roots of the spinal cord of mice [21]; their short-term stimulation induces a long-lasting increase in the presynaptic level of calcium (Ca^2+^) ions and potentiates glutamate release into the synaptic gap [22]. In turn, activation of NMDA glutamate receptors in spinal dorsal horns is needed in order to trigger central sensitization [23, 24, 25, 26].

The spinal flexion reflex was chosen as a nociceptive reaction that occurs at the spinal cord level and whose mechanism involves TRPV1 ion channels and NMDA glutamate receptors. The tail flick test reproducing this reflex [20, 27] allows one to assess the ability of NMDA receptor antagonists to affect TRPV1 activation-induced changes in the animals’ sensitivity to thermal stimulation. VR-/- mice (lacking TRPV1 ion channels) are known to produce an abnormal response to thermal pain stimulation. The C fibers in VR-/- mice are characterized by a lower threshold of the response to thermal stimulation, while the latency of the tail flick response in the tail immersion test at water temperatures of 50 and 52°C (but not 46 and 48°C) and animals’ response in the hot plate test at temperatures of 52.5, 55, and 58°C (but not 50°C) is statistically significantly increased [14]. Therefore, in our experiment (the tail flick test), thermal stimulation was performed by exposing the animals’ tails to thermal radiation, with the temperature gradually increased from 51 to 61°C (during 15 s).

A single intraperitoneal administration of 20 mg/kg BCTC (a TRPV1 ion channel antagonist) was found to significantly increase the latency of the tail flick response in mice – by 36.4% – compared to the control group; the maximum possible effect (MPE) was 15.09% (Table 1). TRPV1 ion channel antagonists are known to possess an analgesic effect [28]. In particular, our findings agree with the data on the efficacy of single intraperitoneal injection of BCTC at doses of 3, 10, and 30 mg/kg for the rat model of thermal hyperalgesia [29].

**Table 1:** The effect of NMDA receptor antagonists (hemantane and MK-801) and the TRPV1 ion channel antagonist (BCTC) on the thermal pain threshold in the tail flick test in ICR mice. Median (Q1; Q3)

Group	Number of mice per group	Latency of tail flick response, s	MPE, %
Control	10	4.40 (3.90; 5.10)	0.00 (-4.72; 6.60)
BCTC, 20 mg/kg	8	6.00 (5.20; 7.35)^*^	15.09 (7.55; 27.83)^*^
Hemantane, 40 mg/kg	8	7.20 (6.30; 10.05)^*^	26.42 (17.92; 53.30)^*^
MK-801, 20 μg/kg	9	4.60 (4.10; 5.20)	1.89 (-2.83; 7.55)
MK-801, mg/kg	9	4.00 (3.80; 4.40)	-3.77 (-5.66; 0.00)

Note: control – saline; MPE – maximum possible effect. * p < 0.05 vs. Control group; Mann–Whitney U test.

The low-affinity NMDA receptor antagonist hemantane, injected intraperitoneally at a dose of 40 mg/kg, increased the latency of the flick tail response in mice by 63.6% compared to the control group; the MPE was 26.42%. No significant intergroup differences between animals that received 20 mg/kg BCTC and 40 mg/kg hemantane were detected (Table 1). Single intraperitoneal administration of 20 and 40 mg/kg hemantane shortened the duration of the pain response to subcutaneous injection of a capsaicin solution into the metatarsal region in mice in a dose-dependent manner; therefore, the 40 mg/kg dose of the agent was used in this study [12].

A single subcutaneous injection of the high-affinity NMDA receptor antagonist MK-801 at a dose of 20 μg/kg (at this dose, it reduced the duration of the capsaicin-induced pain behavior in mice [12]) and a larger dose (0.5 mg/kg) had no significant effect on the thermal pain threshold in the tail flick test in mice (Table 1). Interestingly, single administration of MK-801, the high-affinity NMDA receptor antagonist, induced both the pronociceptive [30] and antinociceptive effects in rats [31].

Single subcutaneous administration of 1 mg/kg capsaicin substantially raised the thermal pain threshold in mice. The latency of the tail flick response to capsaicin administration was 67.4% longer than that in control group mice that had received solvents (saline + saline/ethanol mixture (9:1, v/v)) (Table 2). The detected effect of capsaicin in the tail flick test in mice is attributed to the ability of this agent to induce long-lasting desensitization of nociceptors [32]. Capsaicin did not increase the thermal pain threshold in mice that had preventively received BCTC (a selective TRPV1 ion channel antagonist) or NMDA receptor antagonists (hemantane and MK-801). The effectiveness of BCTC administered to mice 30 min prior to a subcutaneous injection of capsaicin was almost identical to that in the group of animals that had received BCTC only. Thus, the latency of the tail flick response in the group "BCTC, 20 mg/kg + capsaicin, 1 mg/kg" was significantly higher (by 38.04%) compared to the control group (group "saline + saline/ ethanol"); the MPE was 16.83%. Hemantane administered at a dose of 40 mg/kg 30 min prior to capsaicin injection increased the latency of the tail flick response in mice by 21.7% compared to the control group; the MPE was 9.62% (Table 2). Although the latency of the tail flick response in animals that had received hemantane prior to capsaicin injection was lower than that in the animals that had been given hemantane only, no significant differences in the MPE were observed in these groups (Table 1 and Table 2). Administration of MK-801 at both doses 30 min prior to capsaicin injection did not increase the thermal pain threshold in mice in the tail flick test (Table 2).

**Table 2:** The effect of NMDA receptor antagonists (hemantane and MK-801) and the TRPV1 ion channel antagonist (BCTC) on the capsaicin-induced increase in the thermal pain threshold in ICR mice. Median (Q1; Q3)

Group	Number of mice per group	Latency of tail flick response, s	MPE, %
Saline + saline/ethanol	11	4.60 (4.50; 4.80)	0.00 (-0.96; 1.92)
Saline + capsaicin, 1 mg/kg	13	7.70 (6.80; 15.00)^*^	29.81 (21.15; 100.00)^*^
BCTC 20 mg/kg + capsaicin, 1 mg/kg	8	6.35 (5.90; 7.45)^*#^	16.83 (12.50; 27.40)^*#^
Hemantane 40 mg/kg + capsaicin, 1 mg/kg	11	5.60 (4.90; 9.20)^*#^	9.62 (2.88; 44.23)^*#^
MK-801 20 μg/kg + capsaicin, 1 mg/kg	14	4.60 (3.90; 4.90)^#^	0.00 (-6.73; 2.88)^#^
MK-801 0.5 mg/kg + capsaicin, 1 mg/kg	13	3.80 (3.40; 4.60)^*#^	-7.69 (-11.54; 0.00)^*#^

^*^p < 0.05 vs. group “Saline + saline/ethanol,”Mann–Whitney U test.

^#^p < 0.05 vs. group “Saline + capsaicin, 1 mg/kg,”Mann–Whitney U test.

In the groups of mice that had received NMDA receptor antagonists (hemantane and MK-801) and BCTC before capsaicin injection, the latency of the tail flick response was significantly lower than that in the group of mice that had received capsaicin and saline (Table 2).

Hence, the thermal pain threshold was significantly lower in the group of animals that had preventively (before capsaicin injection) been administered the selective TRPV1 ion channel antagonist (BCTC) or NMDA receptor antagonists (hemantane and MK-801) than that in mice that had received capsaicin and saline. This demonstrates that NMDA receptor antagonists and BCTC (a TRPV1 ion channel antagonist) exhibit similar activities. These agents suppressed the effect of capsaicin on TRPV1 ion channels that causes their desensitization and, therefore, significant increase in the thermal pain threshold.

One of the functions of TRPV1 is to get involved in thermoregulation through central and peripheral mechanisms [33, 34, 35]. Systemic administration of cap saicin leads to a rapid transient decline in body temperature, which is brought about by hypothalamus-triggered vegetative reactions, such as vasodilation and hypersalivation [14, 36].

Glutamate receptors in the raphe pallidus nucleus (RPa) mediate the thermogenesis of brown adipose tissue induced by the activation of dorsomedial hypothalamic neurons: microinjections of NMDA or kainic acid into RPa increase the temperature of brown adipose tissue in rats [37]. Preventive administration of LY 235959, a selective NMDA receptor antagonist, weakens hyperthermia induced by icilin (AG-3-5), an agonist of TRPM8 and TRPA1 channels, in rats [38].

**Fig. 1:**
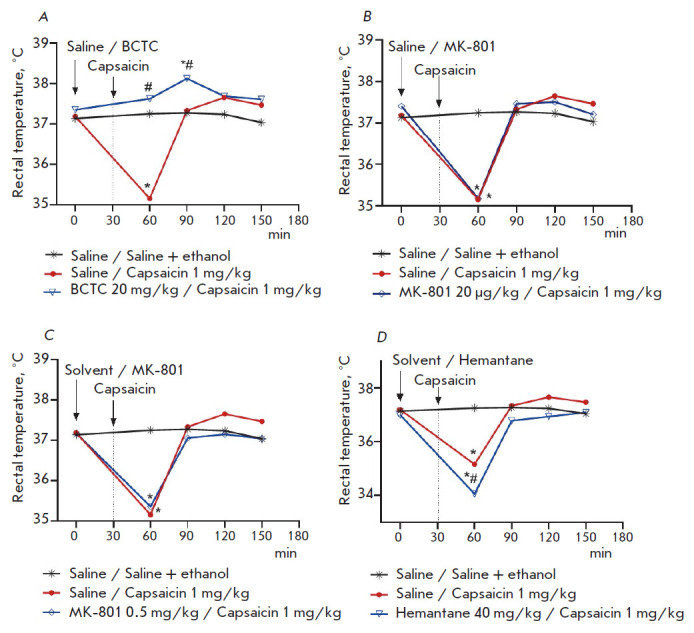
Effect of the TRPV1 antagonist BCTC and NMDA receptor antagonists hemantane and MK-801 on the capsaicin-induced decrease in the rectal temperature in mice. * *p * < 0.05 compared to the group "Saline / Saline + ethanol", the Newman–Keuls test. # *p * < 0.05 compared to the group "Saline / Capsaicin 1 mg/kg", the Newman–Keuls test

In our experiment, a single subcutaneous administration of 1 mg/kg capsaicin to mice induced transient hypothermia, which was observed 30 min after the injection: the rectal temperature decreased by 2°C compared both to its background value (before capsaicin injection) and its value in the control group of mice that had received solvents: saline + saline/ethanol mixture (9:1, v/v). The rectal temperature returned to normal 60 min after capsaicin administration (Fig. 1).

**Fig. 2:**
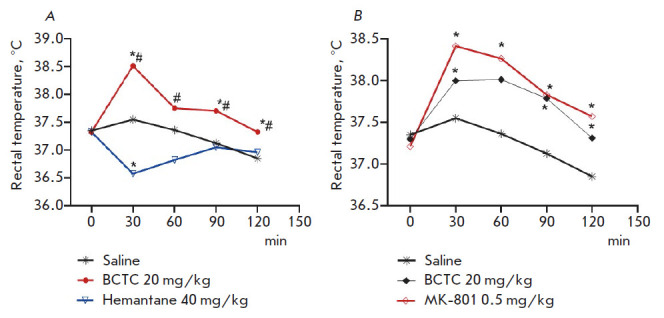
Effect of the TRPV1 antagonist BCTC and NMDA receptor antagonists hemantane and MK-801 on the rectal temperature in mice. * *p * < 0.05 compared to the group "Saline", the Newman–Keuls test. # *p* < 0.05 compared to the group "Hemantane 40 mg/kg", the Newman–Keuls test

A preventive single intraperitoneal dose (20 mg/kg) of BCTC, the selective TRPV1 ion channel antagonist, which is capable of penetrating the BBB [18], suppressed the hypothermic effect of capsaicin. In mice that had sequentially received BCTC and then capsaicin (subcutaneously), the rectal temperature at 30 and 60 min post-injection was significantly higher than that in the animal group receiving capsaicin and saline (by 2.5 and 0.8°C, respectively) (Fig. 1A). Like other TRPV1 ion channel antagonists, BCTC induces hyperthermia, whose formation mechanism remains unclear [32]. Thus, in our experiment, a significant increase in the rectal temperature (compared to the control group of animals) was observed in mice that had received a single intraperitoneal dose (20 mg/kg) of BCTC 30, 90, and 120 min post-injection: by 1, 0.6, and 0.5°C, respectively (Fig. 2A).

A preventive single-dose administration of noncompetitive NMDA receptor antagonists (the high-affinity MK-801 and low-affinity hemantane) did not suppress the hypothermic effect of capsaicin that was observed 30 min after its injection. The rectal temperature in mice that had received the high-affinity NMDA receptor antagonist MK-801 at doses of 20 μg/kg and 0.5 mg/kg prior to capsaicin injection was 35.18 and 35.36°C, respectively, after the injection; in the animals in the group "Capsaicin", the rectal temperature was 35.16°C (Fig. 1B,C). Meanwhile, similarly to BCTC, MK-801 induced hyperthermia in mice at both doses (Fig. 2A,B). The rectal temperature in animals that had received the high-affinity NMDA receptor antagonist at a dose of 0.5 mg/kg significantly exceeded that in the control group of mice, from minutes 30–120 of follow-up; at a dose of 20 μg/kg, 30, 90, and 120 min after administration of MK-801 (Fig. 2B). It is known that MK-801 can induce both hyperthermia, when administered to rats at doses up to 1.2 mg/kg [39, 40], and hypothermia, when its dose is increased to 3 mg/kg [40]. In our study, the observed elevation of the rectal temperature in mice in response to the injection of the high-affinity NMDA receptor antagonist MK-801 may have to do with its dopamine-positive effect. It has been demonstrated for rat striatal synaptosomes that both the noncompetitive NMDA receptor antagonist MK-801 and the competitive NMDA receptor antagonist (+/-)-CPP (3-(2-carboxypyperazin-4-yl)- propyl-1-phosphonic acid) inhibit dopamine reuptake [41]. The dopaminergic system plays a crucial role in body temperature regulation in rats; agonists of D1- and D2-dopaminergic receptors induce hyperthermia in rats [42].

Single-dose preventive intraperitoneal administration of hemantane (40 mg/kg) 30 min prior to capsaicin injection significantly potentiated capsaicin-induced hypothermia: hemantane significantly decreased the rectal temperature in mice by 1.1°C compared to the group "Capsaicin" 30 min after administration of the TRPV1 agonist (Fig. 1D). Meanwhile, when administered intraperitoneally as a single dose (40 mg/kg) to intact animals, hemantane significantly reduced the rectal temperature by 1°C 30 min post-injection; 60 min after hemantane administration, the rectal temperature of mice rose to a value that did not significantly differ from the rectal temperature in the control group (Fig. 2A). Therefore, capsaicin administered 30 min after hemantane injection increases the duration of the hypothermic effect of hemantane to 60 min. After this period (60 min after capsaicin injection), there was no significant difference in rectal temperature between animals that had received hemantane and the control group or the group "Capsaicin" (Fig. 1D).

It was found earlier that single-dose intraperitoneal administration of 20 mg/kg hemantane reduces the levels of serotonin and its metabolite, 5-hydroxyindolacetic acid, in the striatum of C57Bl/6 mice [43]. Therefore, the detected hypothermic effect of hemantane would be apparently attributed to its effect on the serotonergic system, since hypothalamic serotonergic neurons control the body temperature homeostasis, while serotonin injection into the thermosensitive anterior hypothalamic area induces hyperthermia [44].

Excitation of the capsaicin-sensitive peripheral nerves (cutaneous somatosensory afferents and afferent vagus nerve fibers in the abdominal cavity) transmitting signals via the polysynaptic pathways into the preoptic area of the hypothalamus that is responsible for thermoregulation is considered to be a potential mechanism of TRPV1-induced hypothermia [35]. Furthermore, capsaicin, when penetrating the BBB [45], can activate the TRPV1 ion channels of hypothalamic neuronal cells and, therefore, affect thermosensitivity [35]. Thus, capsaicin injection into the preoptic area of the hypothalamus of rats leads to a rapid decline in body temperature; repeated injections of this TRPV1 agonist decrease its intensity [46].

Preliminary administration of NMDA receptor antagonists suppressed the capsaicin-induced increase in the threshold of pain sensitivity in the tail flick test reproducing the spinal flexion reflex in mice and shortened the duration of their response (paw licking) to the injection of a capsaicin solution into the metatarsal region in our earlier study [12]. Intradermal injection of capsaicin into a the paw in rats induced phosphorylation of the NR1 subunit of the NMDA receptors in the neurons of the dorsal horns of the spinal cord and the spinothalamic tract catalyzed by protein kinase A (PKA)- and PKC, which was detected 30 min after capsaicin injection [47, 48]. According to the reported facts of the functional interplay between NMDA receptors and TRPV1 ion channels, we put forward a hypothesis that preliminary administration of NMDA receptor antagonists MK-801 and hemantane would reduce the severity of capsaicin-induced hypothermia by weakening nerve impulse transmission from the periphery (in particular, in the spinal cord) to the preoptic area of the hypothalamus. This, however, did not occur: preliminary administration of NMDA receptor antagonists to the animals did not prevent transient capsaicin-induced hypothermia. Therefore, in the mechanism of transient capsaicin-induced hypothermia in mice, we uncovered no functional interplay between TRPV1 ion channels and NMDA receptors that would be similar to that detected in the experiments aiming to assess the pain response in mice in the tail flick test or the duration of the response in mice when the studied TRPV1 agonist was injected into the metatarsal region [12]. Therefore, our data prove that when administered systemically, capsaicin, a selective TRPV1 ion channel agonist, can penetrate the BBB and act on the neurons in the preoptic area of the hypothalamus, thus affecting thermosensitivity.

## CONCLUSIONS

It has been established in the tail flick test reproducing the spinal flexion reflex in mice that preventive administration of noncompetitive NMDA receptor antagonists (the high-affinity MK-801 and low-affinity hemantane) and the selective TRPV1 ion channel antagonist BCTC inhibits the increase in the ther mal pain threshold induced by capsaicin, a TRPV1 agonist. Taking into account the interplay between NMDA receptors and TRPV1 ion channels at the periphery, the effect observed in the tail flick test in mice can be attributed to the effect exhibited by the tested compounds on afferent innervation. Further studies are needed to evaluate this interplay at the CNS level.

Single-dose subcutaneous injection of capsaicin induces transient hypothermia in mice, and preliminary administration of BCTC, a selective TRPV1 ion channel antagonist, but not the noncompetitive NMDA receptor antagonists MK-801 and hemantane, this effect. Our findings prove that there can be a functional interplay between NMDA receptors and TRPV1 ion channels in the capsaicin-induced antinociceptive response, but this interplay is absent in the case of transient capsaicin-induced hypothermia, whose mechanism is attributed to hypothalamus-triggered vegetative reactions.

## Abbreviations

MK-801: (5S,10R)-(+)-5-methyl-10,11-dihydro-5H-dibenzo[a,d]cyclohepten-5,10-imine hydrogen maleate
BCTC: 4-(3-chloro-2-pyridinyl)-N-[4-(1,1-dimethylethyl)phenyl]-1-piperazinecarboxamide
